# Ultrasound imaging of the thenar motor branch of the median nerve: a cadaveric study

**DOI:** 10.1007/s00330-017-4882-0

**Published:** 2017-06-07

**Authors:** David Petrover, Jonathan Bellity, Marie Vigan, Remy Nizard, Antoine Hakime

**Affiliations:** 1 Centre Imagerie Medicale Bachaumont Paris Centre (IMPC Bachaumont-Blomet Ramsay GDS), 6 rue Bachaumont, 75002 Paris, France; 2 Service de chirurgie orthopédique, Hôpital Lariboisière, AP-HP, 2 rue Ambroise Paré, 75475 Paris Cedex 10, France; 3 Association pour la Recherche en Chirurgie de l’Epaule et du Coude (ARCEC), 20 rue Laffitte, 75009 Paris, France

**Keywords:** Carpal tunnel release surgery, Anatomy, Ultrasound, Median nerve, Dissection

## Abstract

**Introduction:**

Anatomic variations of the median nerve (MN) increase the risk of iatrogenic injury during carpal tunnel release surgery. We investigated whether high-frequency ultrasonography could identify anatomic variations of the MN and its thenar motor branch (MBMN) in the carpal tunnel.

**Methods:**

For each volar wrist of healthy non-embalmed cadavers, the type of MN variant (Lanz classification), course and orientation of the MBMN, and presence of hypertrophic muscles were scored by 18-MHz ultrasound and then by dissection.

**Result:**

MBMN was identified by ultrasound in all 30 wrists (15 subjects). By dissection, type 1, 2 and 3 variants were found in 84%, 3%, and 13% of wrists, respectively. Ultrasound had good agreement with dissection in identifying the variant type (kappa =0.9). With both techniques, extra-, sub-, and transligamentous courses were recorded in 65%, 31%, and 4% of cases, respectively. With both techniques, the bifid nerve, hypertrophic muscles, and bilateral symmetry for variant type were identified in 13.3%, 13.3%, and 86.7% of wrists, respectively. Agreement between ultrasound and dissection was excellent for the MBMN course and orientation (kappa =1).

**Conclusion:**

Ultrasound can be used reliably to identify anatomic variations of the MN and MBMN. It could be a useful tool before carpal tunnel release surgery.

**Key Points:**

• *Ultrasound can identify variations of the motor branch of the median nerve.*

• *Ultrasound mapping should be used prior to carpal tunnel release surgery.*

• *All sub-, extra-, and transligamentous courses were accurately identified.*

• *Type 3 variants (bifid nerve), hypertrophic muscles, and bilateral symmetry were accurately identified.*

## Introduction

**Fig. 1:**
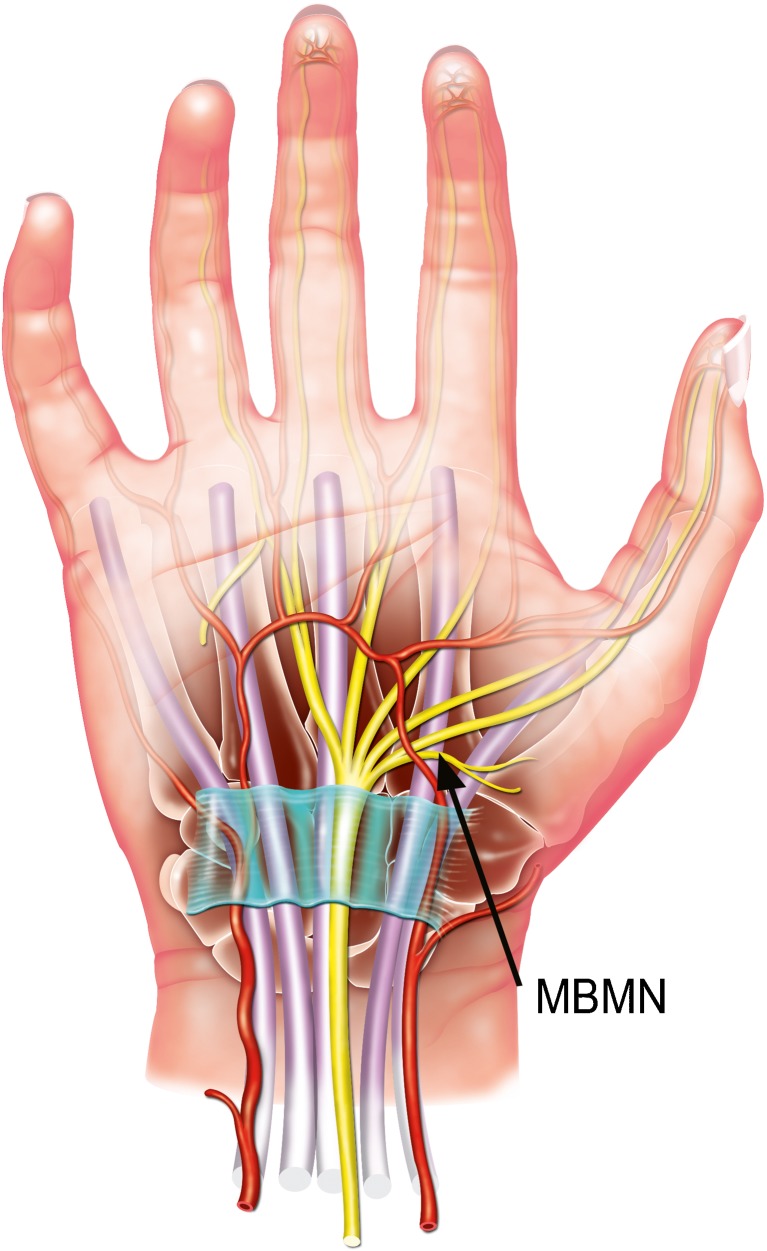
Schematic of the anatomy of the wrist. Standard anatomy. Black arrow: classic variation [median nerve type 1 with an extraligamentous radial course of the motor branch of the median nerve (MBMN)]. Figure provided courtesy of Virginie Denis

Surgery for carpal tunnel syndrome involves sectioning the transverse carpal ligament to release the compressed median nerve. One of the main challenges of this surgery is avoiding damaging small structures, such as the thenar motor branch of the median nerve (Fig. 1), which exists in a number of anatomical variations [1–3]. Some authors report that over 50% of hands exhibit deviations from the standard anatomy [2]. The thenar motor branch supplies the thenar muscles. It provides motor innervation to the abductor pollicis brevis, opponens pollicis, and superficial part of the flexor pollicis brevis. This recurrent branch is called the “million dollar nerve” because of the litigation-related compensation that may occur if it is transected resulting in loss of function of the hand muscles.

As we know of few clinical or electrophysiological signs that denote anatomical variations of the median nerve, surgeons have relied on visualisation during surgery to avoid iatrogenic injury. Open carpal tunnel release surgery lends itself well to the visualisation of the anatomical structures, but it is associated with sizeable scars and lengthy recovery periods [4]. Endoscopic techniques are less invasive and are associated with faster and easier recoveries than open wrist surgery. They, however, are associated with a higher risk of nerve injury due to the restricted field of vision of the endoscope and the inability to anticipate anatomic variations [5, 6]. Continuous sonography combined with minimally invasive percutaneous carpal tunnel release [7–9] enables physicians to demarcate superficial soft tissues and to identify very small anatomic and pathologic details before and during surgery. This relatively new technique relies on the idea that ultrasound can accurately map small anatomical variations.

Few studies have investigated the reliability of ultrasound in the identification and characterisation of the median nerve and its motor branch. In one recently published study, ultrasound was used successfully to identify the thenar motor branch in a cohort of ten cadaveric limbs. Anatomic variations, however, were not formally evaluated [10]. In this article, we present the results of a cadaveric study in which we specifically evaluated the concordance between ultrasonography and dissection in the identification of anatomical variations of the median nerve and its thenar motor branch.

## Material and methods

Approval from the anatomic donations department was obtained prior to the study and the assigned IRB number was COS-RGDS201701002. High-frequency ultrasonography was used to describe the anatomy of the median nerve and its thenar motor branch in the healthy volar wrists of non-embalmed cadavers.

A Hitachi Noblus ultrasound scanner (Hitachi Medical Systems Europe, Zug, Switzerland), a mobile system with an 18-MHz probe, was used. The subject hand was examined in the supine position, and the probe was positioned over the distal wrist crease. The median nerve was identified and then scanned distally until the vertical segment of the thenar motor branch, a vertical, longitudinal hypoechogenicity, was located. All ultrasound examinations were realised by one musculoskeletal radiologist (DP) with 15 years of experience in musculoskeletal ultrasound.

Dissection was then performed by a hand surgeon (JB) who was blinded to the results of the ultrasonography. The incision was made longitudinally along the axis of the palmaris longus tendon. The superficial palmar fascia was dissected to expose the flexor retinaculum. The median nerve was then released and the different branches were dissected distal to proximal.

For each wrist, anatomic parameters were evaluated by ultrasonography and by dissection. They were classified according to the Lanz classification [single thenar motor branch (type 1); accessory branches distal to the flexor retinaculum (type 2); high-division of the median nerve/bifid (type 3); accessory branches proximal to the flexor retinaculum (type 4)] [1]. In addition, the course of the thenar motor branch relative to the carpal tunnel ligament (extra-, sub-, trans-, and preligamentous), orientation of the thenar motor branch (anterior, ulnar, radial), and presence or absence of a hypertrophic muscle overlying the TCL were determined.

### Statistics

Imaging and dissection results were analysed using descriptive statistics. Continuous variables were presented as mean and standard deviation and categorical variables as *n* (%). The agreement between ultrasonography and dissection was calculated by the Cohen’s kappa coefficient (95% confidence interval). Statistical analyses were performed using SAS version 9.4 (SAS Institute Inc., Cary, NC, USA).

## Results

Both wrists of nine healthy cadaveric males and six healthy cadaveric females were included in the study. The mean age at death was 74.8 ± 7.4 years. No signs of scarring or past trauma to the wrists were visible. No additional clinical information was available.

**Fig. 2:**
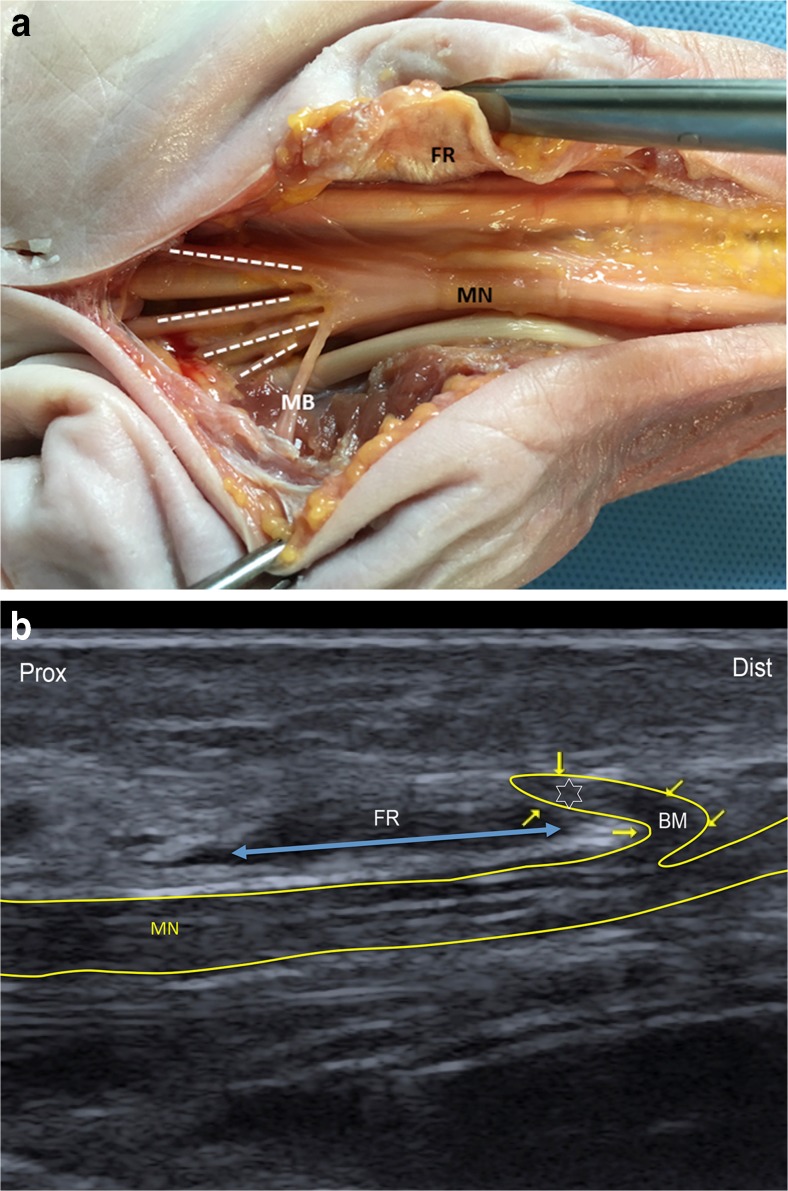
Type 1 anatomic variations of the median nerve and extraligamentous thenar motor branch. **A**) Type 1 Lanz median nerve with an extraligamentous radial thenar motor branch in an un-embalmed cadaveric specimen (anatomic view). The flexor retinaculum was dissected on its radial side. FR = flexor retinaculum; MB = thenar motor branch; MN = median nerve gives rise to the palmar digital nerves (dotted lines). **B**) Type 1 Lanz median nerve with an extraligamentous radial thenar motor branch (ultrasound image). Correlative ultrasound view of the median nerve in the same cadaveric specimen. Median nerve is scanned distally along its long axis (MN); the dorsal to palmar course of the MB (star) around the FR (double arrow) may be detectable as a region of vertically oriented hypoechogenicity (arrow). The thenar motor branch arises distal to the flexor retinaculum and then runs a retrograde course to reach the thenar muscles

**Fig. 3:**
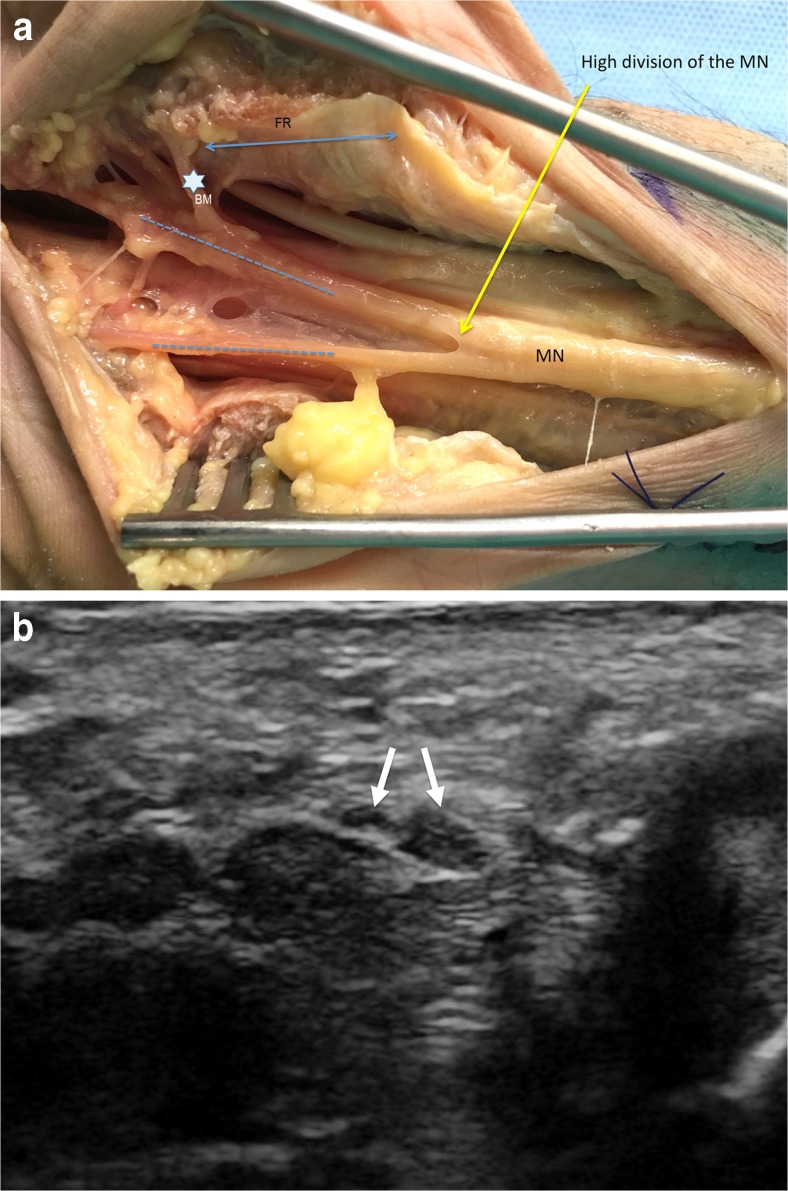
Type 3 anatomic variation of the median nerve. **A**) Lanz type 3 median nerve with a high division (bifid median nerve) in an un-embalmed cadaveric specimen (anatomic view). The flexor retinaculum was dissected on its radial side. FR = flexor retinaculum (double arrow); MB = motor branch (star); MN = median nerve gives rise to the palmar digital nerves (dotted lines). **B**) Lanz type 3 median nerve with a high division (bifid median nerve) in an un-embalmed cadaveric specimen. Ultrasound (US) short axis image of the corresponding cadaveric specimen demonstrating the division into two branches (white arrows) of the MN

**Fig. 4:**
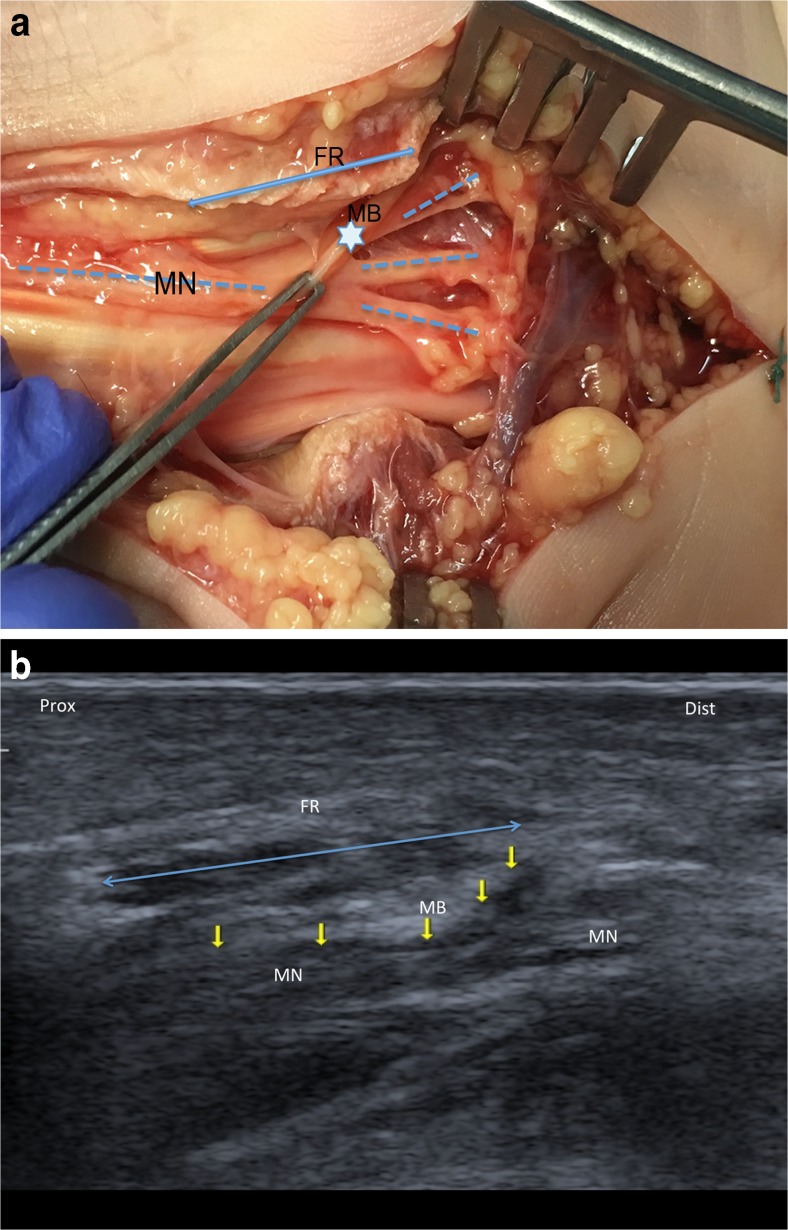
Type 1 anatomic variations of the median nerve and subligamentous thenar motor branch. **A** Type 1 Lanz median nerve with a subligamentous thenar motor branch in an un-embalmed cadaveric specimen (anatomic view). The flexor retinaculum was dissected on its radial side. FR = flexor retinaculum; MB = thenar motor branch; MN = median nerve gives rise to the palmar digital nerves (dotted lines). The thenar motor branch arises within the carpal tunnel under the flexor retinaculum. **B** Type 1 Lanz median nerve with a subligamentous thenar motor branch (ultrasound image). Median nerve is scanned distally along its long axis (MN). The dorsal to palmar course of the MB (star) around the FR (double arrow) may be detectable as a region of vertically oriented hypoechogenicity (arrow). The thenar motor branch arises within the carpal tunnel under the flexor retinaculum and then runs a retrograde course to reach the thenar muscles

By dissection, type 1, 2, and 3 variants were found in 83.4%, 3.3%, and 13.3% of wrists, respectively (Table 1) (Figs. 2 and 3). Four wrists had a bifid median nerve (Fig. 3). The majority of the courses of the thenar motor branch were extraligamentous (65.4%). A subligamentous course was found in eight wrists and a transligamentous course in one wrist (Figs. 2, 4). In 10 out of 15 subjects, bilateral wrists exhibited the same median nerve variant type and thenar motor branch course. Similarly, in other studies, 30-40% of patients did not have identical motor branch origins, numbers, and/or courses [3, 11, 14].

**Table 1:** Anatomic characteristics of the median nerve and its thenar motor branch in 30 healthy cadaveric wrists (15 subjects)

		Ultrasonography	Dissection
Variant type, *n* (%)^a^			
	Type 1	26 (86.7)	25 (83.4)
	Type 2	0	1 (3.3)
	Type 3/bifid	4 (13.3)	4 (13.3)
	Type 4	0	0
Course of the thenar motor branch, *n* (%)			
	Extraligamentous	17 (65.4)	17 (65.4)
	Subligamentous	8 (30.8)	8 (30.8)
	Transligamentous	1 (3.8)	1 (3.8)
	Preligamentous	0	0
Site of branching of the thenar motor branch, *n* (%)			
	Radial	24 (80)	24 (80)
	Anterior	6 (20)	6 (20)
	Ulnar	0	0
Accessory muscle, *n* (%)			
	Absent	26 (87.7)	26 (87.7)
	Present	4 (13.3)	4 (13.3)

^a^Variant type was defined according to Lanz et al., with type 1 being defined as variations in the course of the single thenar motor branch, type 2 as presence of accessory branches of the medial nerve at the distal carpal tunnel, type 3 as high division of the median nerve, and type 4 as accessory branches proximal to the flexor retinaculum

The median nerve and its thenar motor branch were identified by ultrasound in all wrists. Results for motor branch orientation and presence of a hypertrophic muscle were in complete concordance between the two techniques (kappa =1). All transligamentous branches were scored correctly by ultrasound. One discrepancy in variant type classification was found. The wrist was classified, by dissection, as a type 2 variant with two thenar motor branches, a transligamentous and a distal accessory extraligamentous branch. By ultrasound, the wrist was scored as a type 1 variant because the distal accessory extraligamentous motor branch was classified as a sensory branch. For variant type, ultrasound was in good agreement with dissection (kappa =0.9, IC 95% = (0.7-1.0)].

Most orientations were radial (80% of wrists), and the remaining six orientations were anterior. Four wrists (2 subjects) had a hypertrophic muscle, and the hypertrophic muscle was associated with an extraligamentous motor branch course.

## Discussion

In this study of 30 healthy cadaveric wrists, we showed that high-frequency ultrasonography can be used successfully to locate the median nerve, its variant type according to Lanz [1], the course and orientation of the thenar motor branch, and the presence of hypertrophic muscles. Extra-, sub-, and transligamentous courses were identified in all cases. In the context of carpal tunnel release surgery, these results suggest that ultrasonography in the hands of an experienced observer can be used reliably to map the median nerve to reduce the risk of iatrogenic injury.

The ability to identify patients with anatomical characteristics that make them more vulnerable to iatrogenic nerve injury, such as transligamentous and subligamentous motor branch courses and anterior orientations, could have a significant impact on surgical approaches. Endoscopic techniques, which have a narrow field of vision and may be less well suited for patients with transligamentous branches, for example, may need to be preceded by careful ultrasound mapping. The use of percutaneous ultrasound carpal tunnel release combined with continuous ultrasound may increase as the value of identifying high-risk anatomical variations before and during surgery is confirmed.

We found that extraligamentous courses were most prevalent (65.4%), followed by subligamentous courses (30.8%) and transligamentous courses (3.8%). Overall, this is consistent with the distribution of courses reported in the recently published meta-analysis of 31 studies, which included healthy cadaveric wrists and wrists undergoing surgery (3918 wrists) [3]. The rate of transligamentous courses that we report is notably lower than the 11% reported in the meta-analysis. It is, however, consistent with the observation that the rate of transligamentous courses varies significantly from study to study (0 to 80%) [3]. Although an association between race and transligamentous courses has been suggested [3, 11], to date, it has not reached statistical significance and no other clinical characteristics that can be used to predict the likelihood of a transligamentous course have been identified. Ultrasound and visualisation during surgery remain the only methods of identifying transligamentous courses.

Discrepancy between US and anatomical dissection was found in only one wrist, which was classified as a type 2 variant with two branches, a transligamentous and a distal extraligamentous branch by dissection, but as a type 1 variant with a transligamentous course by ultrasound. As this was the only example of a type 2 variant, larger studies would be needed to determine whether high-frequency ultrasonography can be used routinely to distinguish between normal anatomic sensory branches and distal extraligamentous motor branches. In the context of carpal tunnel release surgery, however, the successful identification of the transligamentous branch by ultrasonography was the important finding as a transligamentous branch has a higher risk of being damaged during surgery.

We found no cases of ulnar orientation, supraligamentous courses, or preligamentous courses of the thenar motor branch. This is consistent with studies that showed a 2-3% rate of ulnar orientations [3, 12] and a 1% rate of preligamentous courses [13]. With a sample of only 30 wrists, the representation of these anatomical orientations was unlikely [1]. However, as all three of these presentations increase the risk of iatrogenic injury during carpal tunnel release, additional studies will be necessary to determine whether high-frequency ultrasonography can be used reliably to identify these variations.

As bilateral carpal tunnel syndrome surgery is quite common, the question of symmetry between wrists becomes important. In this study, 40% of subjects were not bilaterally identical. These data support the concept that data from one wrist cannot be extrapolated to the contralateral wrist and that ultrasonography needs to be performed on both wrists prior to surgery.

Lastly, the presence of hypertrophic muscles has been associated with variations of the thenar motor branch course [3, 13, 15, 16]. In the Al-Qattan 2010 study, all transligamentous and preligamentous courses were associated with hypertrophic muscles [13], and in the Green and Morgan 2008 study, 93% of hands with hypertrophic muscles had an anomalous motor branch [15]. Herein, our one case of a transligamentous course was not associated with a hypertrophic muscle; conversely, 100% of wrists with hypertrophic muscles had extraligamentous motor branch courses. However, as our study was performed in healthy wrists rather than in wrists with carpal tunnel syndrome, significant differences between patient populations may exist. In fact, some studies have suggested that carpal tunnel syndrome may be associated with certain anatomical variations such as bifid nerves and subligamentous courses of the thenar motor branch [1, 3, 17]. Finally, we do not think the use of a mobile ultrasound system instead of a high-end system could influence the results.

### Limitations

Inter-observer reliability was not evaluated. As a result, the level of expertise and training necessary to identify the small motor branches by high-frequency ultrasound reliably and reproducibly was not assessed. The limited number of specimens may not take into account all possible anatomical variations. The scans were all performed by an experienced musculoskeletal radiologist and some expertise and training may be needed for evaluation of the thenar motor branch.

## Conclusion

High-frequency ultrasonography was accurate in the identification of anatomic variations of the median nerve, thenar motor branch, and hypertrophic muscles. Detecting high-risk anatomical variations prior to and/or during carpal tunnel release surgery could help reduce the risk of iatrogenic injury.
